# Efficiency of Imazapic Degradation: an Assessment of LacMeta Treatments Utilizing Whole Cell

**DOI:** 10.1007/s00284-026-04760-1

**Published:** 2026-02-23

**Authors:** Natália Sarmanho Monteiro Lima, Elisângela Soares Gomes-Pepe, Flavio Vinicius Crizostomo Kock, Luiz Alberto Colnago, Pedro Luis da Costa Aguiar Alves, Eliana Gertrudes de Macedo Lemos

**Affiliations:** 1 Department of Agricultural, Livestock and Environmental Biotechnology, Faculty of Agricultural and Veterinary Sciences (FCAV), Sao Paulo State University (UNESP), Jaboticabal, 14884-900 São Paulo State Brazil; 2 Molecular Biology Laboratory, Institute for Research in Bioenergy (IPBEN), Jaboticabal, 14884-900 São Paulo State Brazil; 3 Agricultural Microbiology Graduate Program at UNESP, Jaboticabal, São Paulo State Brazil; 4 Sección Química-Departamento Académico de Ciencias, Pontificia Universidad Católica del Perú, San Miguel, Lima, 15088 Peru; 5 Embrapa Instrumentation, São Carlos, 13560-970 São Paulo State Brazil; 6 Department of Biology, Sao Paulo State University (UNESP/FCAV), Jaboticabal, Sao Paulo, Brazil

## Abstract

The extensive use of herbicides such as imazapic, from the imidazolinone class, raises environmental concerns due to its persistence and toxicity in ecosystems and subsequent crops. Enzymatic bioremediation emerges as a sustainable alternative for the mitigation of these contaminants. This study investigated the potential of the metagenomic laccase, LacMeta, expressed in *Escherichia coli* BL21 (DE3), to degrade imazapic using a whole-cell approach. LacMeta expression was optimized with CuSO₄, which proved to be four times more effective than IPTG. The *E. coli* + LacMeta cells demonstrated high tolerance to the herbicide, maintaining cell viability even at high doses (350 g/ha). Notably, the enzymatic activity of LacMeta was not inhibited by imazapic; on the contrary, it was stimulated, reaching a specific activity nearly three times higher in the presence of the herbicide compared to the control. Degradation was confirmed by UV-Visible spectroscopy, which showed the disappearance of imazapic’s characteristic peaks (200–280 nm) over 15 days. ¹H-NMR and FTIR analyses corroborated the degradation, indicating structural changes in the herbicide molecule, particularly in the aromatic ring region (signals at 8.25 and 8.50 ppm). Phytotoxicity assays with lettuce seeds (*Lactuca sativa*) confirmed that treatment with the LacMeta-containing supernatant cell free significantly reduced the toxicity of imazapic in the soil. The results demonstrate that LacMeta has high potential for the bioremediation of imazapic, and the whole-cell approach represents a promising and cost-effective strategy for the decontamination of environments impacted by this herbicide.

**Supplementary Information:**

The online version contains supplementary material available at 10.1007/s00284-026-04760-1.

## Introduction

The use of agricultural pesticides is extremely important for the preservation of the health of cultivated areas and their byproducts, avoiding loss of productivity. These compounds can naturally degrade through physical, chemical, and biochemical processes, but owing to the stability and solubility of some pesticides, they can persist in the environment for an extended period [1]. Pesticide contamination can occur in three regions: the soil surface, aeration zone/vadose zone, and groundwater or saturated zone [2]. Owing to the complexity of the soil, the degradation of pesticides depends on various factors, such as pH, temperature, humidity, and vegetative cover [3]. Biodegradation of pesticides by microorganisms is an efficient means of removing toxic compounds from the environment, as these organisms have the capacity to transform and produce end products with low or no toxicity [4]. The use of enzymes for bioremediation has become increasingly common, as some of these catalysts exhibit a greater degree of promiscuity in relation to their action on substrates, enabling them to act on different molecules with structural similarities [5].

Laccases are enzymes found in various organisms; they contain copper ions in their structure and are known to degrade various compounds, such as dyes, phenols in general, and aromatic amines. Laccases are currently referred to as “green catalysts” since they do not require the use of hydrogen peroxide for oxidation reactions; instead, they use molecular oxygen as the electron acceptor and transform it into water [6, 7]. These enzymes are well known since they have demonstrated potential for the degradation of various pesticides, such as tebuconazole (fungicide) [8], diuron [9] and paraquat (herbicides) [10] and chlorpyrifos (inseticide) [11].

Imazapic acid (±)-2-(4-isopropyl-4-methyl-5-oxo-2-imidazolin-2-yl)-5-methylnicotinic acid is a representative of this class and has been used in peanut cultivation worldwide because of its high efficiency in controlling weeds related to this crop [12]. In soybean and corn crops, it is associated with another imidazolinone (imazapyr) [13, 14]However, its use in successive crops has revealed negative impacts on the productivity of the subsequent crop, and the presence of imazapic residues in the soil and water bodies has already been confirmed; therefore, studies related to the removal/degradation of imazapic compounds are highly important [15]. Additionally, it has a half-life of approximately 44 days and exhibits low adsorption potential, which poses a potential risk of groundwater contamination in the region [16, 17]. Some studies involving microorganisms for the bioremediation of imidazolinones have been conducted and have yielded satisfactory results, such as the use of *Acinetobacter baumannii* for the treatment of imazamox [18] and *Botryosphaeria rhodina* for imazaquin [19], and in this latest study, it was reported that laccases may be involved in the treatment of this herbicide.

Lacmeta is a laccase of metagenomic origin that has been characterized as having the potential to degrade dyes [20]. Recent studies have indicated the potential of LacMeta laccase in the degradation of fentin hydroxide and triphenylmethane dyes [21]. The whole-cell approach involves the use of the heterologous host microorganism *Escherichia coli* BL21 (DE3), which it transformed with a recombinant vector containing LacMeta. Although previous studies have shown that this enzyme has the potential to degrade imazapic, several questions remain, such as where this degradation occurs according to the structure of the compound and whether the compound generated from degradation is toxic. By integrating spectroscopic analyses (UV-Vis, FTIR, and ^1^H-NMR), this study not only proposes a possible novel degradation pathway for imazapic but also reinforces the potential of LacMeta as a versatile biocatalyst in sustainable strategies for agricultural soil remediation.

## Materials and methods

### Obtaining the Inoculum and Expression of LacMeta

LacMeta was an enzyme initially prospected from the soil metagenome of a eucalyptus plantation within a polyketide cluster [22]. Subsequently, the gene encoding the putative enzyme was cloned and the enzyme was characterized [20]. The *E. coli* (DE3) cells containing the LacMeta gene (NCBI code MN974156). were stored in a -80 °C ultrafreezer with 20% glycerol. For the preparation of the preinoculum, a single colony from a solid LB medium culture was inoculated into 50 mL of liquid LB medium and cultured for 16 h at 37 °C and 200 rpm. The inoculum was prepared with 5% preinocula, added to 1 L of liquid LB medium supplemented with 50 µg/ml kanamycin, and induced with 0.25 mmol/L CuSO_4_ after the OD600 reached 0.4–0.6.

### The Effect of the Herbicide Imazapic on the Growth of *E. coli* + LacMeta

After induction with CuSO_4_, doses equivalent to 350, 175, and 150 g/ha imazapic commercial product (Plateau^®^-BASF) were added to each inoculum. Every 24 h, the OD600 nm and number of colony-forming units (CFUs) were measured. The assay was conducted in a growth chamber with rotation at 150 rpm at 30 °C for 15 days. The calculation for the concentration of the commercial product in the medium was:

$$\:Product\:per\:liter\:of\:solution=\:Dose\:(g/ha)/\:Volume\:(L/ha)$$

### LacMeta-Enzymatic Activity Under the Influence of Imazapic

To assess the effect of the tested dose on enzyme activity, aliquots were taken from each dose during the 15-day treatment. For each aliquot, protein extraction was carried out with 20 mmol/L Tris-HCl buffer, 300 mM NaCl, and pH 7.5. The cells were disrupted via ultrasonication (Branson Sonifier 250, Branson, Connecticut, USA), with a cycle ratio of 20% and cycles of 10 s. Laccase enzyme activity was measured using 1 mmol/L ABTS and sodium citrate buffer at pH 4.0 at 37 °C. Protein quantification was performed via the Bradford method [23] All the experiments were conducted in triplicate, and the controls were subjected to the same parameters without the addition of the enzyme. One unit of enzymatic activity was defined as 1 µmol of ABTS released per minute per mg of protein under previously established standard assay conditions. To verify the expression pattern of LacMeta under these conditions, protein electrophoresis was performed with SDS‒PAGE.

### Assessment of Pesticide Degradation Via UV‒Visible Molecular Spectroscopy

For the analysis of possible degradation spectra imazapic standard (Sigma Aldrich, Saint Louis, Missouri, USA) were used. The concentrations used were in accordance with the percentage of the active ingredient (a.i) within the commercial product ( c.p), 350 g/ha c.p (245 g/L a.i), 175 g/ha c.p (122,5 h/L a.i) and 150 g/h c.p (105 g/L a.i). Readings were taken at intervals up of 15 days. UV‒Visible spectroscopy was employed in the spectrophotometric reading range of 200–900 nm with a 5 nm interval, using a UV‒Vis Multiscan GO spectrophotometer (Thermo Fisher Scientific, Waltham, MA, USA). Assays were conducted with the whole-cell.

### Nuclear Magnetic Resonance (^1^H-NMR) and Fourier Transform Infrared (FTIR) Analysis

The enzymatically treated samples of imazapic were incubated at 30 °C with agitation at 150 rpm and then subjected to high-resolution 1 H-nuclear magnetic resonance (NMR) analysis in deuterated water. This analysis was performed via a Bruker Avance III 600 (14 T) spectrometer at the NMR Laboratory of Embrapa Instrumentation, which uses 5 mm BBI probes with a Z gradient at 25 °C. The spectra were processed via Topspin software (Bruker), in which the signals were filtered with a 0.5 Hz line exponential filter. After Fourier transformation, the spectra underwent manual phase and baseline corrections. For FTIR, each sample was prepared in potassium bromide (KBr) as a pellet at a 1:99 sample to KBr ratio, and was recorded by the ABB FTLA 2000 − 100 (ABB Co., Quebec, Canada) at a resolution limit of 16 cm^−1^.

### Phytotoxicity of the Degraded Compounds

The imazapic, the assessment was performed in polypropylene boxes (Gerbox^®^) containing a 2:1 ratio of soil to sand, where the herbicide was applied at a dose of 150 g/ha, and the supernatant was inoculated the next day. After 15 days (the predetermined treatment time), the lettuce seeds were sown. The germination velocity index and dry matter weight of the aboveground and root parts, germination percentage, and imazapic mixture were evaluated.

### Statistical Analysis

All experiments were carried out with statistical replicates (triplicates). The data were analyzed by analysis of variance (ANOVA) with Tukey’s multiple comparisons test.

## Results

### LacMeta-induced Resistance of *E. Coli* Cells To Imazapic

One of the main advantages of heterologous protein production is the high productivity of the enzyme of interest. However, several purification steps and the use of inputs are often needed, making the process relatively more time-consuming and expensive. Therefore, for this assay, the *E. coli* BL21 (DE3) clone containing the LacMeta gene was used. A preliminary assay to identify the most efficient expression method was conducted, where LacMeta expression was performed via two inducers, IPTG (0.1 mmol/L), the most common inducer of heterologous proteins, and CuSO_4_ (0.25 mmol/L), which also acts as an inducer of LacMeta and other laccases (patent-deposited BR 10 2020 026200 9). When the enzyme activity of laccase was compared with that of each inducer, the value was 83.9 for 319 U, which was four times greater than that observed for IPTG. Therefore, copper induction was chosen for the subsequent assays. Interestingly, LacMeta was also secreted into the supernatant; therefore, in the subsequent analyses, we chose to use the CFS in the following analyses (Table S1).

**Fig. 1:**
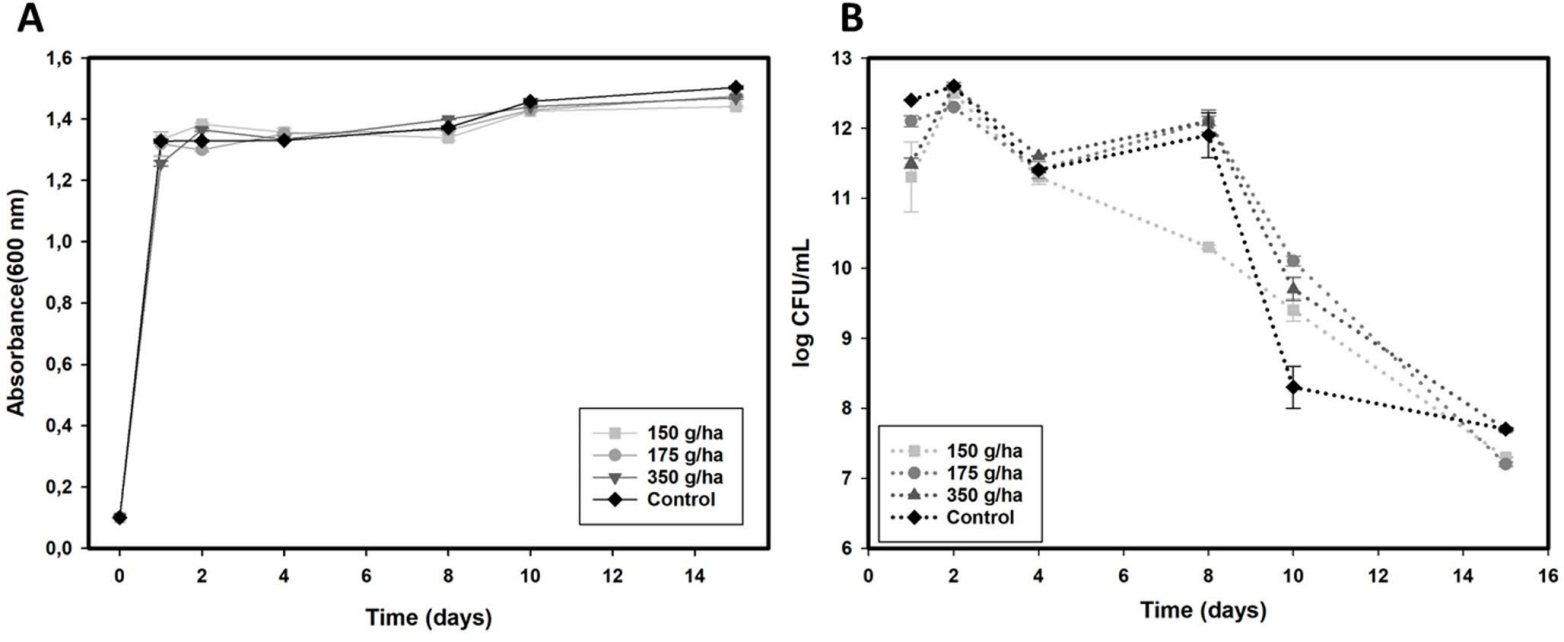
Effect of imazapic on the growth of *E. coli* + LacMeta. **A**) Bacterial growth curve (O.D. 600 nm); **B**) CFU/mL (colony forming units per milliliter). Treatment time: 15 h. Cultures were incubated for 15 h at 37 °C in LB medium containing 350 g/ha imazapic or control (without herbicide). Error bars indicate standard deviation (*n* = 3)

Despite monitoring optical density (OD 600 nm) being one of the easiest ways to track the toxicity of certain compounds in bacterial cell development, CFU analysis is considered the most suitable methodology to confirm this toxicity. It allows for the detection of the number of remaining live and viable individuals in a sample over time, making it more reliable for detecting the lethal effects of different treatments [24]. The absorbance data obtained from the treatment groups were not significantly different from those of the control group (Fig. 1A), indicating that cell multiplication occurred even at a high dose of imazapic (350 g/ha). Similar results were obtained for CFU/mL. Although a greater number of viable cells were detected in the first 24 h (one day), by the end of the 15-day experiment, the values for the control and the highest dose were exactly the same (7.7 log CFU/mL). These values were not significantly different from those obtained for the other doses of 150 and 175 g/ha, with 7.3 and 7.2 log CFU/mL, respectively (Fig. 1B). The results of this study demonstrated that the dosis of imazapic did not significantly affect *E. coli* + LacMeta cells.

### Enzymatic Activity of Laccases Exposed To Imazapic

A crucial factor for degradation efficiency is verification of whether the xenobiotic compound acts as an inhibitor of microbial enzymatic activity or even if the heterologous protein maintains stable activity throughout the treatment. To evaluate the activity of the herbicide imazapic, at each assessment point, enzymatic activity with ABTS and total protein quantification were performed. In the first 24 h, the treatments with 350 and 175 g/ha had higher specific activities than did the control, especially the 350 g/ha treatment, which had nearly triple the activity of the control (Table 1). This stimulation of enzymatic activity is intriguing, as some compounds, such as pesticides, can act as enzyme inhibitors, which did not occur in this situation. In fact, the highest dose was responsible for the highest specific activity. Even at the end of the treatment, despite the presence of imazapic, the 150 g/ha sample was the most enzymatically active. The analysis via SDS‒PAGE revealed that LacMeta continued to be overexpressed throughout the assay, further reinforcing the ability of the enzyme to be used in the treatment/bioremediation of this compound (Figure S1).

**Table 1:** Protein concentration and laccase activity throughout Imazapic treatment with the ABTS substrate (1 mmol L^− 1^), 20 mmol L^− 1^ sodium citrate buffer, pH 4,0, 37 °C for 30 min ±

	Sample											
	Control			350 g/ha			175 g/ha			150 g/ha		
*Time* *(h)*	*Protein (mg)*	*Total Activity* *(U)*	*Specific Activity (U/mg)*	*Protein (mg)*	*Total Activity (U)*	*Specific Activity (U/mg)*	*Protein (mg)*	*Total Activity (U)*	*Specific Activity (U/mg)*	*Protein (mg)*	*Total Activity (U)*	*Specific Activity (U/mg)*
24	17.2 ± 0.1	11.1 ± 0.1	0.6 ± 0.05	14.8 ± 0.7	26.9 ± 0.4	1.8 ± 0.07	11.6 ± 0.6	9.4 ± 0.3	0.8 ± 0.07	11.6 ± 0.07	19.1 ± 0.03	1.0 ± 0.03
48	10.2 ± 0.04	12.2 ± 0.02	1.2 ± 0	15.3 ± 0.2	26.0 ± 0.1	1.7 ± 0.03	14.4 ± 0.4	13.6 ± 0.2	0.9 ± 0.04	14.4 ± 0.1	18.3 ± 0.2	1.8 ± 0.3
96	16.4 ± 0.02	8.2 ± 0.04	0.5 ± 0	16.7 ± 0.2	12.8 ± 0.5	0.7 ± 0.02	17.3 ± 0.6	9.8 ± 0.3	0.6 ± 0.03	17.3 ± 0.2	10.0 ± 0.4	0.5 ± 0.01
192	16.2 ± 0.01	10.9 ± 0.01	0.7 ± 0.06	16.8 ± 0.7	8.7 ± 0.2	0.5 ± 0.02	17.7 ± 0.3	22.1 ± 0.3	1.3 ± 0.01	17.7 ± 0.2	13.9 ± 0.3	0.8 ± 0.02
240	20.2 ± 0.01	9.7 ± 0.01	0.5 ± 0.02	28.1 ± 0.5	9.4 ± 0.7	0.3 ± 0.04	22 ± 0.2	9.4 ± 0.1	0.4 ± 0.02	22 ± 0.4	9.9 ± 0.3	0.5 ± 0.01
360	14 ± 0.2	7.6 ± 0.2	0.5 ± 0.01	19.3 ± 0.2	9.9 ± 0.1	0.5 ± 0.01	18.8 ± 0.04	9.9 ± 0.04	0.5 ± 0.01	18.8 ± 0.01	11.6 ± 0.01	0.6 ± 0.03

### Imazapic Degradation with UV‒visible

**Fig. 2:**
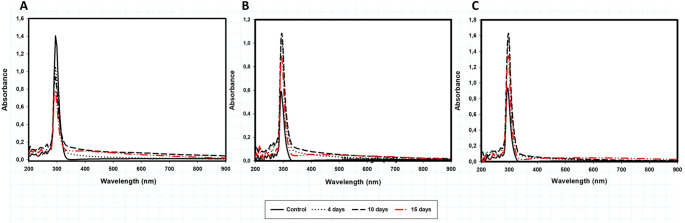
UV‒visible spectra of Imazapic at doses of (**A**) 350 g/ha, (**B**) 175 g/ha and (**C**) 150 g/ha and its metabolites after treatment with LacMeta for 15 days. Readings were taken from 200–900 nm

UV‒visible analysis revealed when imazapic was degraded by LacMeta. However, after 15 days of treatment, this peak disappeared, indicating that the products formed by degradation with absorbance in this region were also degraded (Fig. 2A, B and C). Both doses tested in this study exhibited the same behavior, in which the absorbance values in this region (200–280 nm) decreased after 10 days, and by 15 days, the region representing the highest incidence of peaks had virtually disappeared for all the treatments. Low-absorbance peaks appeared only at the highest concentration of the herbicide. The growth data demonstrated that *E. coli* + LacMeta was capable of continuing to degrade imazapic over 15 days.

### NMR Spectroscopy and FTIR

**Fig. 3:**
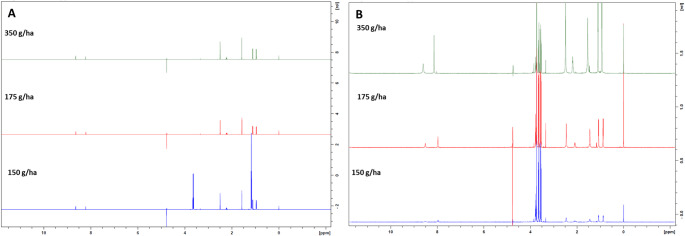
^1^H-NMR spectra of the compounds. Imazapic at doses of 350 g/ha, 175 g/ha and 150 g/ha and its metabolites were treated with LacMeta for 15 days. (**A**) Spectrum of the controls without treatment. (**B**) Spectrum after treatment

**Fig. 4:**
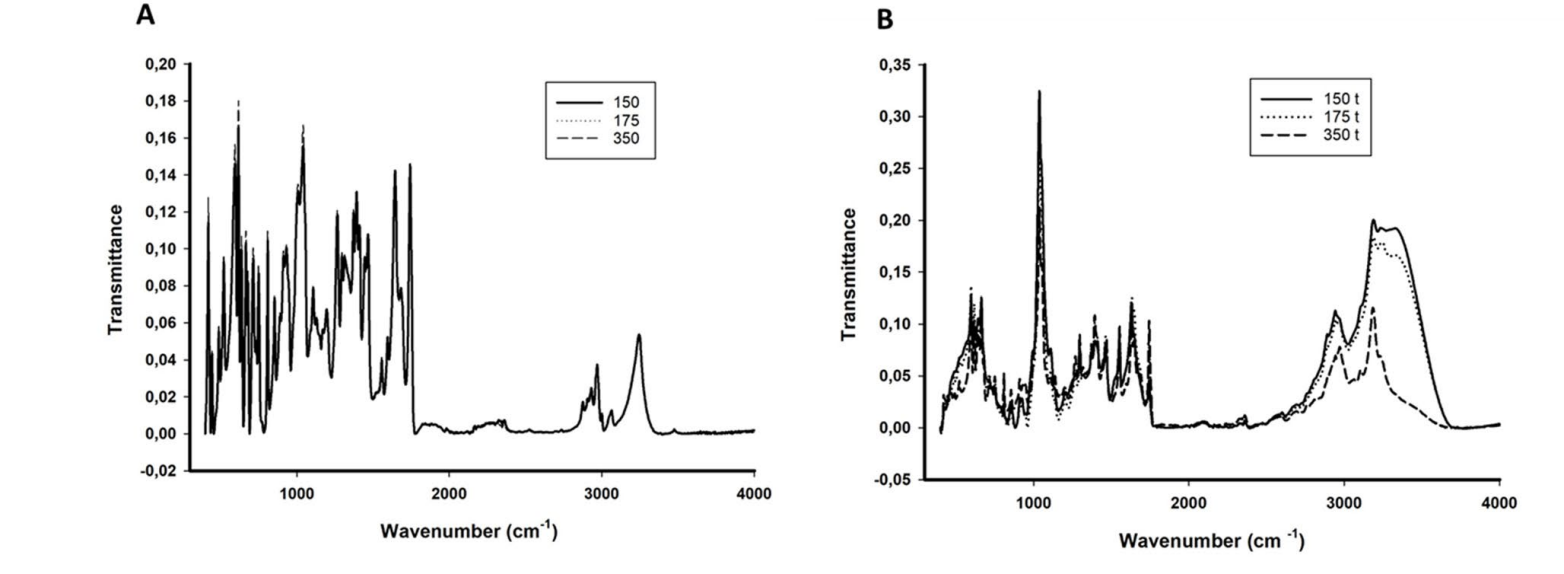
Fourier transform infrared (FTIR) spectrum of imazapic at concentrations of 350 g/ha, 175 g/ha and 150 g/ha and its metabolites treated with LacMeta for 15 days. (**A**) Spectrum of controls - without treatment. (**B**) Spectrum after treatment

Imazapic presented characteristic spectra, with two close singlets (8.25 and 8.50 ppm), followed by other singlets, a doublet at 1.5 ppm and a singlet at 0.9 pmm (Fig. 3C). These spectra indicate that herbicide degradation occurred mainly in the 8 ppm region and in the 3.15 ppm region, which had a singlet before enzymatic action. The presence of a quartet was observed after treatment (Fig. 4D). The FTIR analyses showed similar behavior, with changes in peak frequency and size in the 400–1770 cm⁻¹ region and the appearance of new peaks between 2488 and 3657 cm⁻¹ (Fig. 4).

### Phytotoxicity Assay

**Fig. 5:**
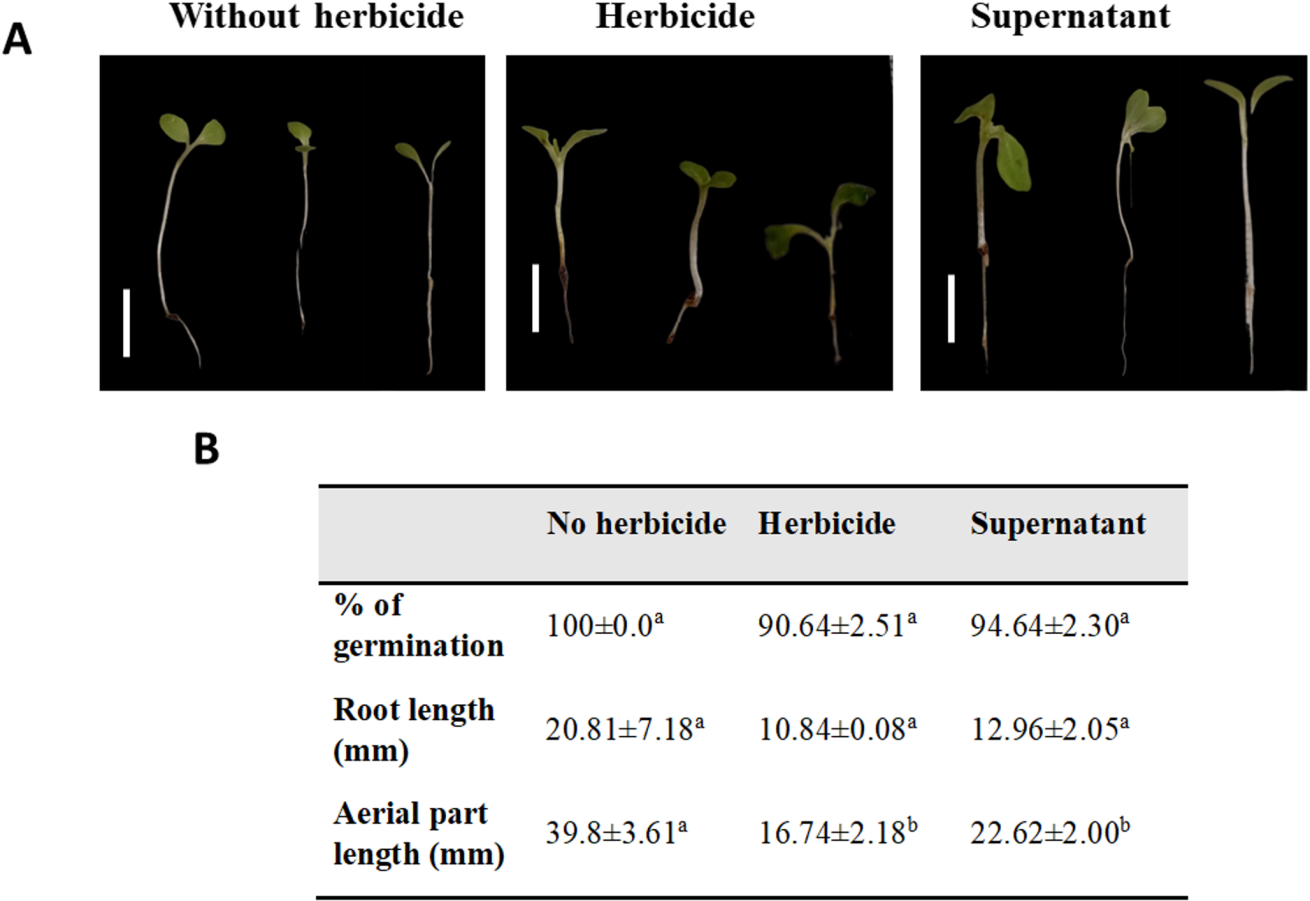
Phytotoxicity assay of imazapic-generated metabolites in lettuce. (**A**) Appearance of 7-day-old seedlings in the presence of water or 150 g/ha imazapic. (**B**) Effects on germination percentage, root length and aerial part length. Control: without imazapic (water). The letters indicate differences between treatments (*p* < 0.05). Scale bars = 1.0

The assay conducted with imazapic aimed to assess the efficiency of the enzyme in the soil (Fig. 5A). When the germination percentage was compared, the difference between the treatments was small, but when the seedling development parameters were evaluated (Fig. 5B), treatment with the enzyme-containing CFS reduced the phytotoxicity of the herbicide.

## Discussion

*E. coli* is known to be a model organism and may or may not have the ability to survive in adverse environments and even with enzymes that help in the degradation of xenobiotics, increasing the survival of these bacteria. Some studies, such as those of Botelho and collaborators [25], who tested 13 herbicides and their effects on the growth of *E. coli* ATCC 25,922, have indicated that this strain is resistant to most herbicides except for Gramoxone^®^ (paraquat). Olchanheski et al. [26] also evaluated the effect of Callisto^®^ (mesotrione) on *E. coli* DH5α, but after 6 h, a decrease in cell viability was observed. The CFU mL^− 1^ of *Rhodococcus erythropolis* were significantly reduced after exposure to atrazine [27]. A common factor in these studies is that the dose used was lower than the recommended dose for field application, and the evaluation time for the effect on the strains was considerably shorter than what we evaluated.

When the degradation mechanism of a compound depends on the action of an enzyme, it is subject to factors that can inhibit the activity of that enzyme, and one of these can be the concentration of the compound to be treated. When exposed to the agricultural pesticide imazapic, the activity of LacMeta surprisingly increased. The catalase activity was affected when *E. coli* K-12 (wild type and mutants) were inoculated together with Gramoxone^®^ after 9 h of treatment [28]. In the literature, other herbicides, including others from the imidazolinone group, have shown potential as inducers of laccases. (Table 2). These findings indicate the strong potential of these enzymes for the removal and transformation of these compounds [29]. Another factor that affects this stability is the fact that LacMeta is a bacterial laccase, and these laccases are generally more stable than fungal laccases [30].

**Table 2:** Effect of herbicides as inducers or inhibitors of microbial and plant Lacase activity

Organism /Origin	Herbicide	Chemical Group	Structure	Effect on activity	Ref.
Metagenomic	Plateau^®^ (Imazapic)	Imidazolinone	C_14_H_17_N_3_O_3_	+	This work
*Botryosphaeria rhodina*	Specter^®^ (Imazaquin)	Imidazolinone	C_17_H_17_N_3_O_3_	+	[19]
*Ganoderma lucidum*	Bentazon (Basagran^®^) e Diuron	Benzothidiazole Phenylurea	C_10_H_12_N_2_O_3_S e C_9_H_10_Cl_2_N_2_O	+	[31]
*Trametes versicolor*	Isoproturon	Urea	C_12_H_18_N_2_O	+	[32]
*Abortiporus biennis*	Paraquat	Bipyridyl	C_12_H_14_Cl_2_N_2_	+	[10]
*Oryza Sativa* ^*#*^	Atrazine	Thiazine	C_8_H_14_ClN_5_	+	[33]

#plant

+inducer

-inhibitors

**Fig. 6:**
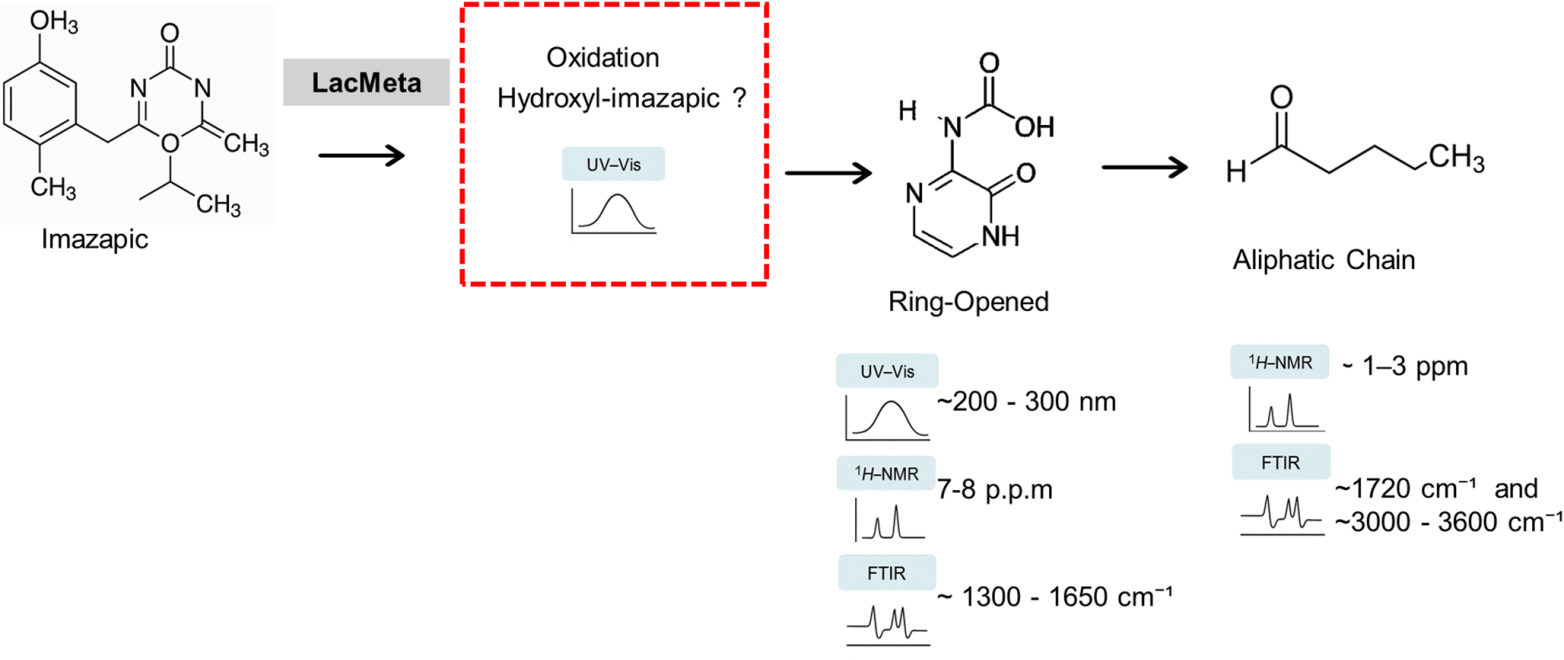
Proposed degradation pathway for the degradation of imazapic by LacMeta using the UV-Vis, H-NMR, and FTIR analysis report

Integrating the data obtained from the UV–Vis, ¹H-NMR, and FTIR analyses, it is possible to infer the degradation events leading to the first proposed pathway for the herbicide imazapic (Fig. 6). The UV‒vis spectrum of the herbicide imazapic displays characteristic peaks in the 200–280 nm range [34–36]. Moreno and collaborators [37] reported that after 20 h of treatment, the spectrum between 270 and 300 nm increased substantially, generating a new peak in this region. Another interesting factor is the peaks after 280 nm and close to 300 nm, which demonstrate the relationship of imidazolinones with copper ions and generate absorbance exactly in that region [37, 38], and as the residues of these herbicides are progressively degraded, this absorbance decreases. The ¹H-NMR and FTIR results suggest successive transformation steps. In the ¹H-NMR spectrum, the disappearance of aromatic signals (δ ≈ 7–8 ppm) accompanied by the emergence of new aliphatic signals (δ ≈ 1–3 ppm) indicates hydroxylation and ring cleavage, resulting in the formation of smaller metabolites containing carboxylic and hydroxyl functional groups. Correspondingly, the FTIR spectrum shows a decrease in the characteristic C = N (~ 1650 cm⁻¹) and aromatic C–N (~ 1300 cm⁻¹) bands, along with the appearance of new absorptions at C = O (~ 1720 cm⁻¹) and O–H (~ 3400 cm⁻¹), consistent with the formation of carboxylic acids, alcohols, and phenolic compounds. In the study by Lima et al. [21]on the action of LacMeta against Malachite Green (MG), the enzyme promoted complete degradation of the dye, as evidenced by the disappearance of proton signals corresponding to aromatic rings in the ^1^H-NMR spectrum.

Similarly, in the experiments with imazapic treated with LacMeta, the loss or shift of aromatic proton signals observed in the ^1^H-NMR spectrum suggests that LacMeta also induced the cleavage of aromatic rings in this herbicide molecule.

The phytotoxicity assays confirmed the efficiency of LacMeta as a bioremediation agent for both compounds. Given that the environment was much more complex (soil), with various variables that could affect the bioremediation process, for imazapic, the best results (UV‒vis) were achieved with the lowest dose (150 g/ha). The most significant results for the imazapic spectrum were the degradation of the peaks corresponding to the aromatic rings (8.5 and 8.25 ppm). This outcome was akin to the LacMeta enzyme mechanism in the case of malachite green dye, in which its aromatic rings were degraded [21]. Despite their low concentrations, these compounds are the most commonly used compounds in crops, such as peanuts, in which 140 g/ha is applied, a value lower than the value evaluated. Furthermore, this usage can lead to factors such as water body contamination [39] and carryover. Residues of imidazolinones, as persistent herbicides, represent a potential for environmental contamination due to their high bioactivity; for this reason, the elucidation of the microorganisms and enzymatic systems involved in their degradation is highly recommended [17]The results were satisfactory, demonstrating that the CFS was able to substantially degrade the herbicide, highlighting the potential of this enzyme. Depending on the type of soil, imazapics may exhibit alterations in phytotoxicity, as elucidated by Vasconcelo and collaborators [15], who evaluated four plant species, and all four showed phytotoxicity, with the severity changing depending on the soil. These results highlight the efficiency of LacMeta in degrading imazapic. However, several factors still need to be elucidated. For example, how does this enzyme behave in different types of soil? How does it perform under varying rainfall conditions? What is the degradation pathway of this compound? This study serves as a reference not only for further research on this enzyme but also for studies involving other enzymes or microorganisms with the potential to degrade agricultural pesticides.

## Conclusions

Research conducted on LacMeta demonstrated its efficacy as a bioremediation agent. The treatment with LacMeta was effective against imazapic, resulting in its degradation, and the herbicide did not exhibit any inhibitory effect on the enzymatic activity. The phytotoxicity assays revealed that, even with partial degradation of the herbicide, the soil test results corroborated the reduction in phytotoxicity. These findings are pioneering for imazapic.

## Supplementary Information

Below is the link to the electronic supplementary material.

Supplementary Material 1

## Data Availability

The data that support the findings of this study are available from the corresponding author upon reasonable request.
