# Supplementary material for: Efficiency of Imazapic Degradation: an Assessment of LacMeta Treatments Utilizing Whole Cell
Source: Curr Microbiol. 2026 Feb 23;83(4):206. doi: 10.1007/s00284-026-04760-1 (PMC12929357; doi:10.1007/s00284-026-04760-1)
Supplement: Supplementary file 1 — Supplementary Material 1 [file 284_2026_4760_MOESM1_ESM.docx]

| Enzyme | Fraction Volume (mL) | Protein (mg/mL) | Total Activity  (units/mg) |
| --- | --- | --- | --- |
| IPTG – extraction-purification* | 8 mL | 1,33 | 1,62 |
| CuSO_4_ -supernatant (Whole cell) | 1. L | 0,96 | 333,69 |

**Table S1.** Comparison of activity and concentration between the methods used

*after size exclusion chromatography


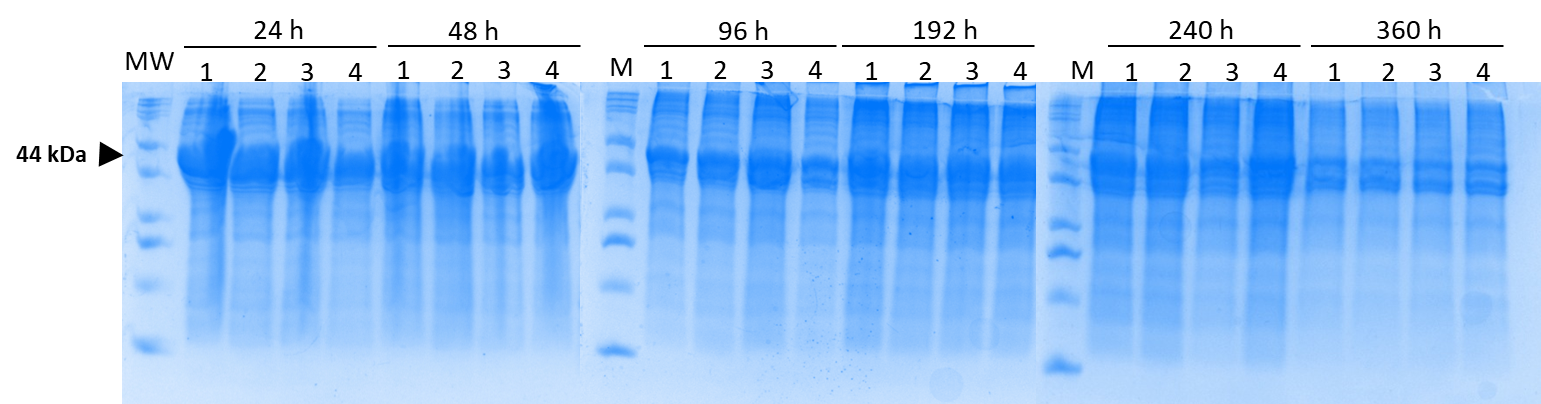


**Figure S1.** SDS-PAGE gel analysis of 10% of *E.coli* + LacMeta samples co-inoculated with imazapic. 1) Control; 2) 350 g/ha; 3) 175 g/ha; 4) 150 g/ha. MW = molecular weight marker.
